# Perceptual Decision Impairments Linked to Obsessive-Compulsive Symptoms are Substantially Driven by State-Based Effects

**DOI:** 10.5334/cpsy.87

**Published:** 2022-05-12

**Authors:** Claire M. Kaplan, Alec Solway

**Affiliations:** 1 Department of Psychology, University of Maryland-College Park, MD 20742, USA; 2 Department of Psychology and Program in Neuroscience and Cognitive Science, University of Maryland-College Park, MD 20742, US

**Keywords:** Obsessive Compulsive Disorder, drift-diffusion model, perception, state vs trait

## Abstract

Computational models of decision making have identified a relationship between obsessive-compulsive symptoms (OCS), both in the general population and in patients, and impairments in perceptual evidence accumulation. Some studies have interpreted these deficits to reflect global disease traits which give rise to clusters of OCS. Such assumptions are not uncommon, even if implicit, in computational psychiatry more broadly. However, it is well established that state- and trait-symptom scores are often correlated (e.g., state and trait anxiety), and the extent to which perceptual deficits are actually explained by state-based symptoms is unclear. State-based symptoms may give rise to information processing differences in a number of ways, including the mechanistically less interesting possibility of tying up working memory and attentional resources for off-task processing. In a general population sample (N = 150), we investigated the extent to which previously identified impairments in perceptual evidence accumulation were related to trait vs stated-based OCS. In addition, we tested whether differences in working memory capacity moderated state-based impairments, such that impairments were worse in individuals with lower working memory capacity. We replicated previous work demonstrating a negative relationship between the rate of evidence accumulation and trait-based OCS when state-based symptoms were unaccounted for. When state-based effects were included in the model, they captured a significant degree of impairment while trait-based effects were attenuated, although they did not disappear completely. We did not find evidence that working memory capacity moderated the state-based effects. Our work suggests that investigating the relationship between information processing and state-based symptoms may be important more generally in computational psychiatry beyond this specific context.

## Introduction

Obsessive-Compulsive Disorder (OCD) is characterized by the presence of perseverative, unwanted, and intrusive thoughts (obsessions) which can trigger excessive urges to perform certain overt actions and covert mental rituals (compulsions). When compared to people with anxiety or unipolar mood disorders, individuals with obsessive-compulsive disorder are less likely to be married, more likely to be unemployed, and more likely to report impaired social and occupational functioning (Abramowitz, Taylor, & McKay, 2009; Torres et al., 2006; Veale & Roberts, 2014). The substantial impact that OCD has on functioning can also be seen in studies showcasing an array of cognitive and behavioral abnormalities in these patients, including impairments in decision making (Foa et al., 2003; Pushkarskaya et al., 2015; Reed, 1976; Sachdev & Malhi, 2005).

**Figure 1:**
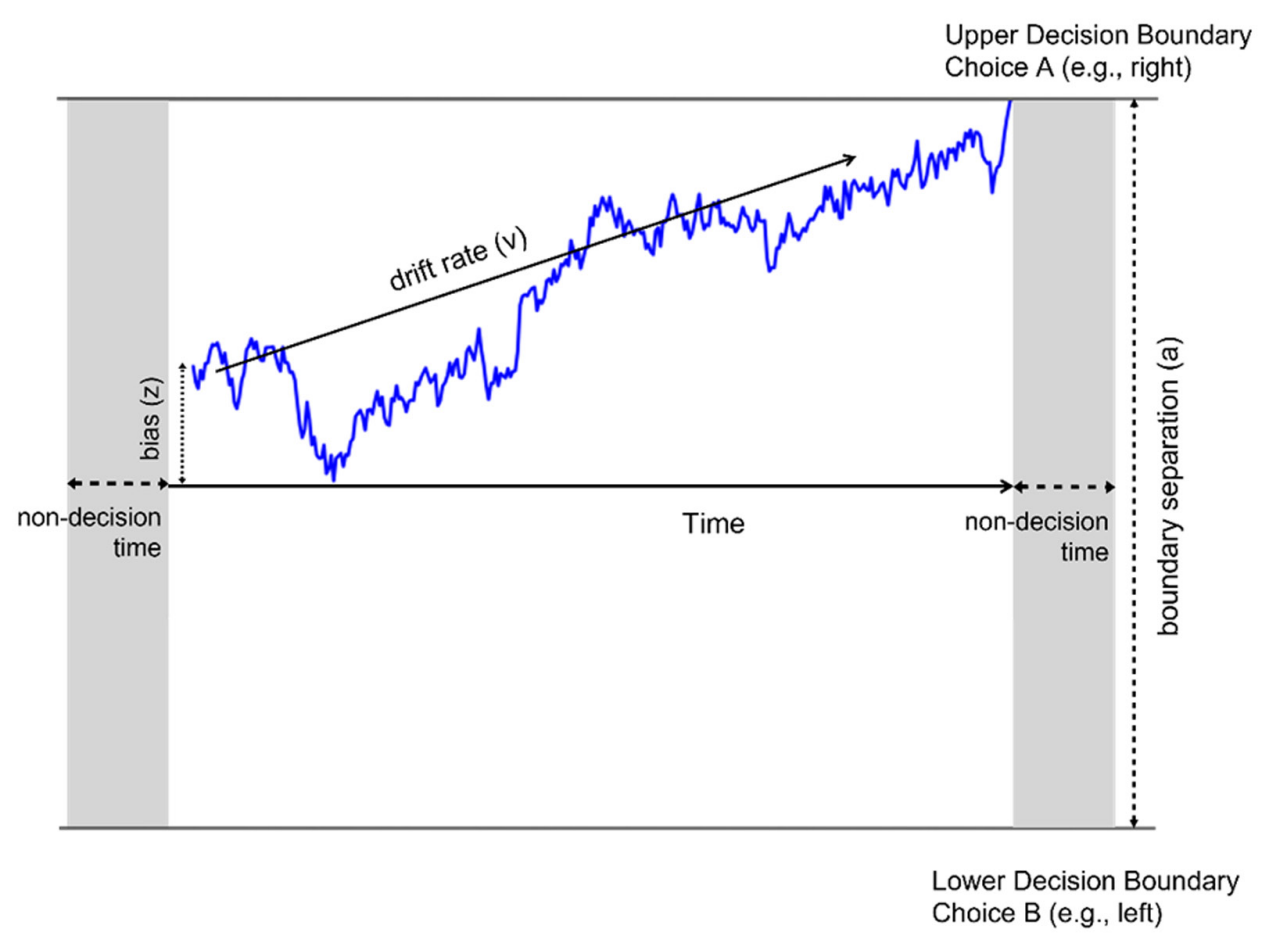
**Example of a DDM Decision Process.** The DDM describes the accuracy and reaction time of decisions using a basic mechanism of evidence accumulation with a drift-diffusion process. In a given trial, an individual will continuously extract noisy sensory evidence from the presented stimulus. This noisy evidence is accumulated over time, pushing a decision variable towards one of two decision boundaries. Once enough evidence has been sampled to push the decision variable across a boundary, a decision is made and the respective response initiated. This process is described by the following parameters: *drift rate*, which represents the rate of evidence accumulation towards either decision boundary; *boundary separation*, which represents the distance between the two decision boundaries; *bias*, which represents a priori bias towards one or another decision boundary at the start of the trial; and *non-decision time*, which represents time spent on decision-independent processing.

The ability to make effective and timely decisions is a cornerstone of healthy human functioning. Computational models have formalized the intuitive idea that decision making involves accumulating evidence for and against the options under consideration until a function of this evidence reaches a threshold (Bogacz, Brown, Moehlis, Holmes, & Cohen, 2006; Smith & Ratcliff, 2004; Townsend & Ashby, 1983). A particularly popular exemplar from this class of models is the drift-diffusion model (DDM), which applies to decisions with two possible outcomes (Ratcliff, 1978; Ratcliff & McKoon, 2008). Each decision in the DDM (***Figure 1***) is modeled as a directed Brownian motion toward an upper or lower decision boundary, representing the accumulation of noisy evidence in favor of one versus the other option. When the accumulated evidence reaches one of the boundaries, the decision is made and the respective response initiated. Several parameters describe this process, including: *drift rate*, which represents the rate of evidence accumulation towards either decision boundary; *boundary separation*, which represents the distance between the two decision boundaries; and *non-decision time*, which represents time spent on decision-independent processing. In the perceptual domain, this model has been frequently studied in the context of the dot motion task, where on each trial the participant sees an array of dots, and has to decide in which direction the majority of them are moving (Gold & Shadlen, 2007; Newsome, Britten, & Movshon, 1989; Ratcliff & McKoon, 2008; Shadlen & Newsome, 1996).

Using the DDM and this well-established perceptual decision task, recent studies have documented slower rates of evidence accumulation (slower drift rates) both in OCD patients and in individuals from the general population reporting high trait levels of symptoms (Banca et al., 2015; Erhan et al., 2017; Hauser, Allen, Rees, & Dolan, 2017; Marton et al., 2019; Solway, Lin, & Vinaik, 2021, but see also Erhan & Balcı, 2017). In addition, these impairments have been found to be larger for easier decisions. Relationships such as this between psychiatric symptoms and latent parameters of computational models are an important area of study within the field of computational psychiatry. However, while studies looking at associations with trait symptom measures are common, relationships with state-based symptoms are usually not investigated. This leaves unclear whether information processing differences captured in computer-based tasks are truly related to longer-term beliefs and behaviors or are more situationally bound. The work looking at OCD-related perceptual impairments has followed this common approach and has focused only on trait measures. In contrast, the importance of investigating state effects in OCD has previously been described in the neuropsychology literature, where it has been suggested that state-dependent impairments may exist and may be epiphenomena of state-based symptoms (Abramovitch, Dar, Hermesh, & Schweiger, 2012; Moritz, Hottenrott, Jelinek, Brooks, & Scheurich, 2012).

Answering these questions has obvious important implications for treatment development. If perceptual impairments are related to longer-term, trait-level symptomatology, then treatment may be directed at rescuing those impairments with the reasonable argument that one might expect the symptoms to thus improve. On the other hand, if perceptual impairments are actually a byproduct of active state-level symptoms, such interventions will likely be ineffective and treatment needs to focus instead on other upstream factors.

Given the correlation between trait and state symptoms, it is important to test whether one or both are related to information processing differences. Our primary goal in the present work was to begin to ask this question for computationally defined perceptual deficits related to OCS. However, similar questions may also be warranted in other domains within the purview of computational psychiatry.

A second question of interest concerned whether differences in working memory capacity moderated drift rate impairments. If impairments are secondary to state-based effects as some have previously suggested (Abramovitch et al., 2012; Moritz et al., 2012), it is possible that they may be reduced in individuals with larger working memory capacities, who have more resources to expend on processing both their symptoms and the task at hand. To ask both sets of questions, we had participants complete the dot motion decision making task as in previous work on perceptual deficits (Banca et al., 2015; Erhan et al., 2017; Hauser et al., 2017; Marton et al., 2019; Solway et al., 2021), the OSPAN task (Turner & Engle, 1989), and trait and state assessments of obsessive-compulsive symptoms.

## Methods

### Participants

Data were collected online using Amazon’s Mechanical Turk (MTurk) (Buhrmester, Kwang, & Gosling, 2016). Eligible participants were at least 18 years of age, had a US billing address, and had a prior task approval rating of at least 95%. Upon study completion, included participants were paid $5 plus a bonus based on overall accuracy in each task (max bonus = $1.00; M = $0.76, SD = 0.19). All participants provided electronic informed consent in accordance with procedures approved by the University of Maryland, College Park Institutional Review Board (#1155349).

Several a priori exclusion criteria were applied to ensure data quality in line with standard practices and suggestions for studies using MTurk (Crump, McDonnell, & Gureckis, 2013; Gillan, Kosinski, Whelan, Phelps, & Daw, 2016). Participants that did not pass a comprehension quiz following the instructions and task demo could not continue. Those that completed the experiment were sequentially excluded if: their total accuracy across all trials of the dot motion task was below 55% (n = 69), their accuracy on the processing component of the OSPAN (mathematics problems) was below 85% (n = 16, in addition to those excluded at the previous step) (Conway et al., 2005), they answered either of two questionnaire catch-items incorrectly (n = 25), or they completed a 12-item catch trial in the OSPAN task without error (to catch cheating; n = 16). Data from 150 participants were included in the analysis (out of 276 subjects who submitted data). Dot motion trials with implausibly fast (<250ms) or unusually slow (>15 sec) reaction times were discarded. See **Supplement** for additional details.

### Tasks

To assess perceptual decision making, we used the well-studied random dot-motion task (RDMT) (Gold & Shadlen, 2007; Newsome et al., 1989; Ratcliff & McKoon, 2008; Shadlen & Newsome, 1996). Participants were asked to determine the primary direction of motion of a dynamic kinematogram consisting of small white dots moving within a circular aperture in the middle of a black screen. Trials consisted of varying levels of motion coherence either leftward or rightward; coherence levels were set to 7.5% (“High uncertainty”), 20% (“Medium uncertainty”), and 45% (“Low uncertainty”), and presented in random order across trials. To assess working memory capacity, we used the well-established Operation-Span (OSPAN) task. Within each trial, participants were shown a series of simple math problems alternating with individual letters. Participants were asked to verify the correctness of each presented math problem, and to remember the letters in the order presented for later recall at the end of the trial. Individual working memory span scores were calculated using the partial-credit unit scoring method, in which participants receive credit for the percentage of items recalled in correct serial position per set—these are then averaged to produce the total partial unit score (for more details, see Conway et al., 2005). See **Supplement** for additional details.

### Questionnaires

Consistent with prior work on the relationship between perceptual decision making and OCD symptoms (Erhan & Balcı, 2017; Hauser et al., 2017), we used the well-validated Padua Inventory-Washington State University Revision (PI-WSUR) (Burns, Keortge, Formea, & Sternberger, 1996) as one assessment of trait-level OCS (Rubio-Aparicio et al., 2017). The PI-WSUR consists of 39-items comprising five symptom-cluster subscales: contamination obsessions and washing compulsions, dressing/grooming compulsions, checking compulsions, obsessional thoughts of harm to self/others, and obsessional impulses to harm self/others. The format of this measure, however, does not lend itself well for an assessment of state-level symptoms during a lab based experiment—the two biggest challenges being that it was not originally designed to consider the impact of symptoms over any specific time frame, and that it is content-specific and includes many instances of obsessions and compulsions which would not be applicable during an experiment (e.g., dressing and grooming). The developers of the PI-WSUR reported total scores for both normative and OCD patient samples (normative: M = 22, SD = 16; patients: M = 55, SD = 17) (Burns et al., 1996).

Another popular, well-validated measure of obsessive-compulsive symptoms is the Yale-Brown Obsessive Compulsive Scale—Self-Report (Y-BOCS-SR)(Baer, Brown-Beasley, Sorce, & Henriques, 1993; Federici et al., 2010; Goodman, 1989; Steketee, Frost, & Bogart, 1996). The Y-BOCS-SR contains 10-items to assess the severity of obsessions and compulsions with five rating dimensions: time spent/occupied, interference with functioning, degree of distress, resistance, and control (i.e., success in resistance). The standard Y-BOCS-SR is designed to assess symptoms over the past week and asks the individual to rate their experience of obsessions and compulsions at large regardless of the specific content of their symptoms (e.g., contamination versus harm). Prior studies using the Y-BOCS-SR have reported total scores for both non-clinical and OCD patient samples (normative: M = 5, SD = 5; patients: M = 21, SD = 5) (Steketee et al., 1996) To assess the severity of state-level obsessions and compulsions (i.e., during the RDMT and OSPAN tasks), we made minor modifications to the Y-BOCS-SR, replacing the wording for each question to indicate the participant consider their symptoms specifically “During the Dots and Math/Letters tasks” (whereas, in the standard version this is specified as “In the past week”). We refer to this version as “Y-BOCS-SR—State”. We asked participants to complete both the standard Y-BOCS-SR and the Y-BOCS-SR—State. See **Supplement** for additional details.

### Hierarchical Drift-Diffusion Modeling of the RDMT

The drift-diffusion model (DDM) is widely used to explain the latent dynamics of two-choice decision-making (Ratcliff, 1978; Ratcliff & McKoon, 2008; Ratcliff & Rouder, 1998; Smith & Ratcliff, 2004). The model has been extensively studied in a number of different contexts, including the dot motion task used here (Gold & Shadlen, 2007; Heekeren, Marrett, & Ungerleider, 2008; Newsome et al., 1989; Ratcliff & McKoon, 2008; Shadlen & Newsome, 1996). Raw aggregate behavioral measures, such as accuracy and reaction time, arise from the decision process described by the model. Considering individual components of processing and model parameters allows for both more specific questions to be asked and increased statistical power (White, Ratcliff, Vasey, & McKoon, 2010). In the current study, we employed hierarchical Bayesian parameter estimation of the basic form of the drift-diffusion model (without across-trial variance, see Lerche & Voss, 2016), using Markov chain Monte Carlo to compute posteriors for group-level parameters while also accounting for individual differences.

All fitted models were specified with the following free parameters: *drift rate* (i.e., rate of evidence accumulation towards either decision boundary), *boundary separation* (i.e., distance between the two boundaries, or amount of evidence necessary to decide), and *non-decision time* (i.e., time spent on decision-independent processing, such as initial perceptual encoding and motor execution). Drift rate and boundary separation were allowed to vary by subject and trial uncertainty level (i.e., dot-motion coherence), and were simultaneously regressed on the variables of interest (questionnaire scores and working memory span). Non-decision time varied by subject only without additional regressors. The DDM also allows for an a priori bias for the drift process starting point relative to the two decision boundaries. All models assumed an unbiased starting point, given that left/right responses in the RDMT were counterbalanced. We fit and present a series of progressively more complete regression models. This approach is not meant to suggest that we advocate comparing across these models. The final model, which includes all regressors, simultaneously asks all of the questions of interest and is the one we defer to for answers. However, given that previous work has looked at trait effects alone, we began by considering trait effects only and tested whether we could replicate this work in the context of previous assumption. We then built up from this starting point. See the **Supplement** for additional details.

### Statistical Analyses

Dependent samples Welch’s t-tests were used to compare the accuracy and reaction times of different trial uncertainty levels in the RDMT. Hierarchical drift-diffusion models were implemented with Stan, using the RStan package in R (R Core Team, 2019; Stan Development Team, 2018). Effects reported are the posterior median value and the median 95% credible interval (CI) of the regression coefficients in our models. We considered an effect “significant” if its median 95% CI did not include 0. To test whether two effects were “significantly” different, we subtracted their posterior distributions from one another and assessed whether the 95% CI of the resultant distribution did not include 0. All independent variables in the regressions were z-scored.

## Results

The study sample comprised 150 adult participants. ***Table 1*** lists the sample’s demographics and characteristics (OSPAN and questionnaire scores).

**Table 1:** Demographics and Characteristics of the Sample.


	TOTAL	MIN–MAX

Age, Years	39 ± 12	19–71

Sex		

Female	73 (49)	–

Male	77 (51)	–

OSPAN	0.88	0.29–1

PI-WSUR		

Total	22 ± 22	0–121

Checking	8 ± 8	0–34

Contamination	9 ± 9	0–38

Grooming	1 ± 3	0–12

Obsessional Impulses	1 ± 4	0–30

Obsessional Thoughts	3 ± 4	0–21

Y-BOCS-SR		

Total	7 ± 6	0–22

Obsessions	4 ± 4	0–13

Compulsions	3 ± 3	0–13

Y-BOCS-SR—State		

Total	3 ± 5	0–18

Obsessions	2 ± 3	0–10

Compulsions	2 ± 2	0–16


Values are mean ± SD or *n* (%). OSPAN, partial unit score for letter recall in the Operation Span task; PI-WSUR, Padua Inventory-Washington State University Revision; Y-BOCS-SR, Yale-Brown Obsessive Compulsive Scale Self Report standard version; Y-BOCS-SR—State, Yale-Brown Obsessive Compulsive Scale Self Report modified to assess state-level symptoms experienced during completion of experimental tasks.

**Figure 2:**
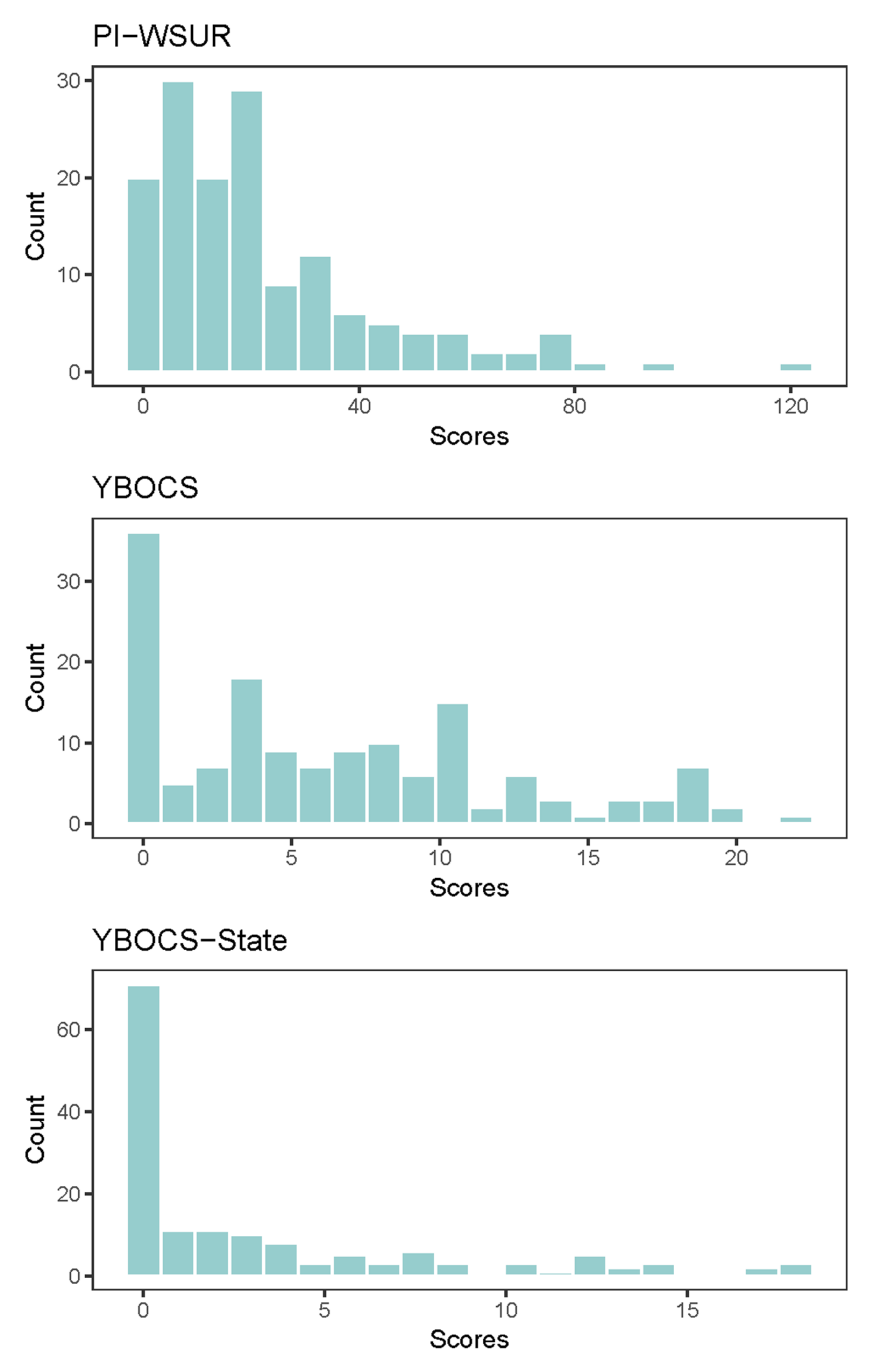
**Distributions of Questionnaire Total Scores.** Distributions of total scores for the PI-WSUR, standard Y-BOCS-SR (“YBOCS”), and our modified Y-BOCS-SR—State (“YBOCS-State) questionnaires. PI-WSUR scores ranged from 0 to 121 (M = 22, SD = 22). YBOCS scores ranged from 0 to 22 (M = 7, SD = 6). YBOCS-State scores ranged from 0 to 18 (M = 4, SD = 5).

Distributions of total scores for the PI-WSUR, standard Y-BOCS-SR, and our modified Y-BOCS-SR—State questionnaires are presented in ***Figure 2***. The PI-WSUR scores ranged from 0 to 121 with a mean of 22 and standard deviation of 22. The standard Y-BOCS-SR scores ranged from 0 to 22 with a mean of 7 and standard deviation of 6. The modified Y-BOCS-SR—State scores ranged from 0 to 18 with a mean of 4 and standard deviation of 5. ***Table 2*** presents the correlation matrix between the questionnaire total scores and OSPAN working memory scores. As an exploratory analysis, we also tested the relationships between these variable and age. There were no significant relationships between participant age and questionnaire total scores, though there was a significant association between age and OSPAN working memory capacity (r = –0.23, t = –2.9, p = 0.005).

**Table 2:** Correlations of WM and Psychometric Questionnaires.


	OSPAN	PI-WSUR	YBOCS	YBOCS-STATE

OSPAN	1	0.029	–0.002	0.13

PI-WSUR	0.029	1	0.477*	0.312*

YBOCS	–0.002	0.477*	1	0.499*

YBOCS-State	0.13	0.312*	0.499*	1


Notes. * = *p* < .001 (fdr-corrected). OSPAN = partial unit score for letter recall in the Operation Span task; PI-WSUR = Padua Inventory-Washington State University Revision; YBOCS = Yale-Brown Obsessive Compulsive Scale Self Report standard version; YBOCS-State = Yale-Brown Obsessive Compulsive Scale Self Report modified to assess state-level symptoms experienced during completion of experimental tasks.

**Figure 3:**
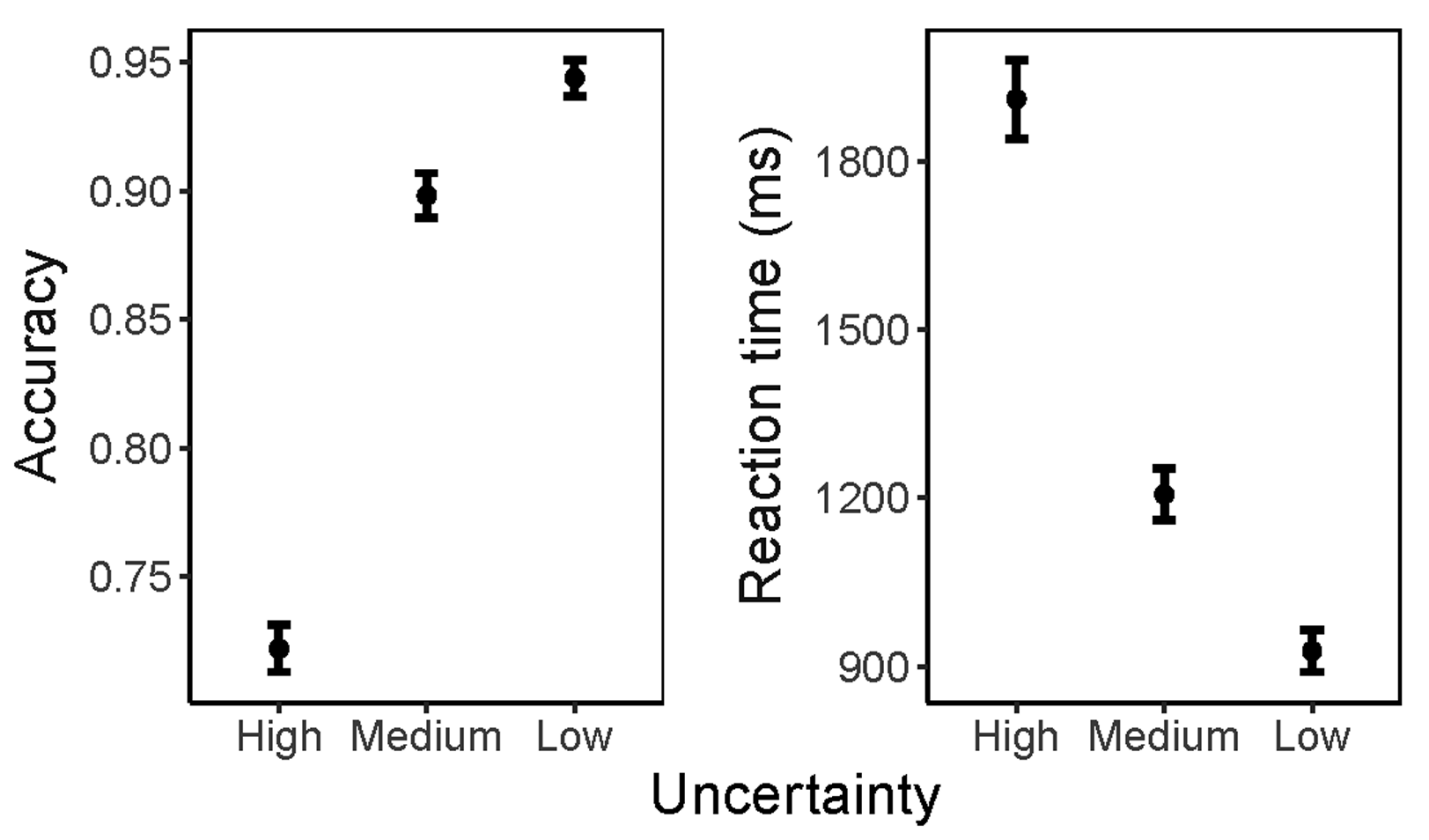
**Difficulty in the RDMT is Modulated by Uncertainty Level of the Motion Stimulus.** Uncertainty level represents motion stimulus coherence (Low, 45% coherence; Medium, 20% coherence; High, 7.5% coherence). Medium uncertainty trials were more accurate than high uncertainty trials (t(149) = 26.7, p = 1.2e–58), and low uncertainty trials were more accurate than medium uncertainty trials (t(149) = 10.6, p = 7.8e–20). Similarly, medium uncertainty trials had shorter reaction times than high uncertainty trials (t(149) = –14.4, p = 6.9e–30), and low uncertainty trials had shorter reaction times than medium uncertainty trials (t(149) = –14.7, p = 6.8e–31).

Basic behavioral data from the RDMT are presented in ***Figure 3***, which demonstrates how the level of uncertainty (coherence) in the dot motion stimulus affects difficulty. Uncertainty levels were as follows: “High” (7.5% coherence), “Medium” (20% coherence), and “Low” (45% coherence). Consistent with expectation, higher uncertainty trials in the RDMT were both less accurate (High – Med: t(149) = –26.7, p = 1.2 × 10^–58^; Med – Low: t(149) = –10.6, p = 7.8 × 10^–20^) and slower (High – Med: t(149) = 14.4, p = 6.9 × 10^–30^; Med – Low: t(149) = 14.7, *p* = 6.8 × 10^–31^).

Applying the drift-diffusion model, there was a consistent main effect of stimulus uncertainty for both drift rate and boundary separation across all regression models: the lower the uncertainty, the faster the drift rate and the smaller the boundary separation. ***Table 3*** lists these parameters and their contrasts for the first model (Model 1, described below), with nearly identical results for all other models.

**Table 3:** Posterior Median and Central 95% Credible Interval for Main Group-level Parameters in Model 1.


	MEDIAN	LOWER	UPPER

**Drift rate**			

*Subject, SD*	0.41	0.37	0.47

*Group, Stim uncertainty level*			

Low	1.80	1.73	1.87

Medium	1.20	1.13	1.27

High	0.41	0.34	0.47

*Group, Diff between uncertainty levels*			

Low – Medium	0.60	0.57	0.63

Medium – High	0.80	0.77	0.82

**Boundary separation**			

*Subject, SD*	0.56	0.50	0.63

*Group, Stim uncertainty level*			

Low	1.93	1.84	2.02

Medium	2.15	2.06	2.24

High	2.45	2.36	2.54

*Group, Diff between uncertainty levels*			

Low – Medium	–0.22	–0.24	–0.20

Medium – High	–0.30	–0.32	–0.28

**Non-decision time**			

*Subject, mean*	0.38	0.36	0.40

*Subject, SD*	0.13	0.11	0.14


Effects are considered significant when the median 95% CI does not include 0. As such, all of the effects and contrasts listed in Table 2 above are significant.

Our first regression model (Model 1) included only the relationship with PI-WSUR score, which has been used in prior studies to characterize OCS-related differences in perceptual decision making (Hauser et al., 2017; Solway et al., 2021), to test whether we could replicate this work. Model 1 demonstrated a significant negative relationship between PI-WSUR score and drift rate for easier trials (i.e. Low and Medium, but not High, uncertainty level), with a greater negative effect at Low compared to Medium uncertainty, and at Medium compared to High uncertainty (Low: β = –0.16 [–0.23, –0.09]; Med: β = –0.07 [–0.13, –0.01]; High: β = –0.02 [–0.09, 0.05]; Difference of Low – Med: β = –0.09 [–0.12, –0.07]; Difference of Med – High: β = –0.04 [–0.06, –0.02]). There were no significant effects of PI-WSUR on decision boundary separation. The posterior median estimates and 95% CIs for all regressors across all models are shown in **Supplemental Table S1**.

To test whether the standard trait-based Y-BOCS-SR (“YBOCS”) similarly captured performance differences, our second model (Model 2) specifically focused on it. Results were similar to Model 1: a significant negative impact of YBOCS score on drift rate in Low uncertainty trials more so than Medium uncertainty trials, and in Medium uncertainty trials more so than High uncertainty trials (Low: β = –0.17 [–0.24, –0.10]; Med: β = –0.06 [–0.13, –0.01]; High: β = –0.01 [–0.08, 0.06]; Difference of Low – Med: β = –0.11 [–0.14, –0.08]; Difference of Med – High: β = –0.05 [–0.07, –0.03]). There were no significant effects of YBOCS on decision boundary separation.

**Figure 4:**
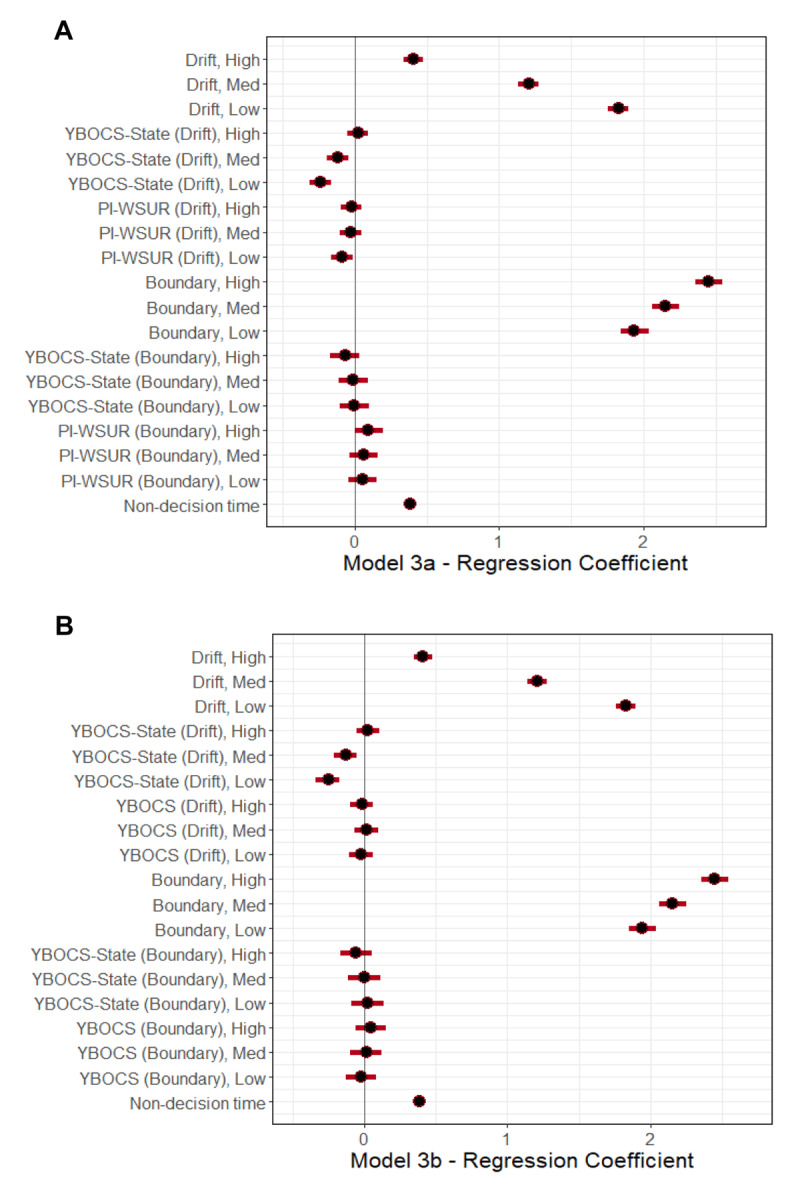
**Posterior Median and 95% CI for Model 3a and Model 3b Regression Coefficients.** Panel (A) shows parameters for Model 3a; Panel (B) shows parameters for Model 3b. “Low” = low uncertainty trials at 45% dot motion coherence; “Med” = medium uncertainty trials at 20% coherence; “High” = high uncertainty trials at 7.5% coherence. For each model, the following parameters are shown for each level of stimulus uncertainty: group level drift rate (“Drift”); effects of subject-level scores on the Y-BOCS-SR—State (“YBOCS-State”), Padua Inventory (“PI-WSUR”), and/or the standard Y-BOCS-SR (“YBOCS”) on drift rate (Drift); group level decision boundary separation (“Boundary”); effects of subject-level scores on the YBOCS-State, PI-WSUR, and/or YBOCS on boundary separation (Boundary). Also shown is the group level mean for non-decision time. The median estimate for each contrast’s posterior distribution is represented by a black dot, and the 95% CI is represented by a red line. An effect is considered significant if the 95% CI does not overlap with 0.

**Figure 5:**
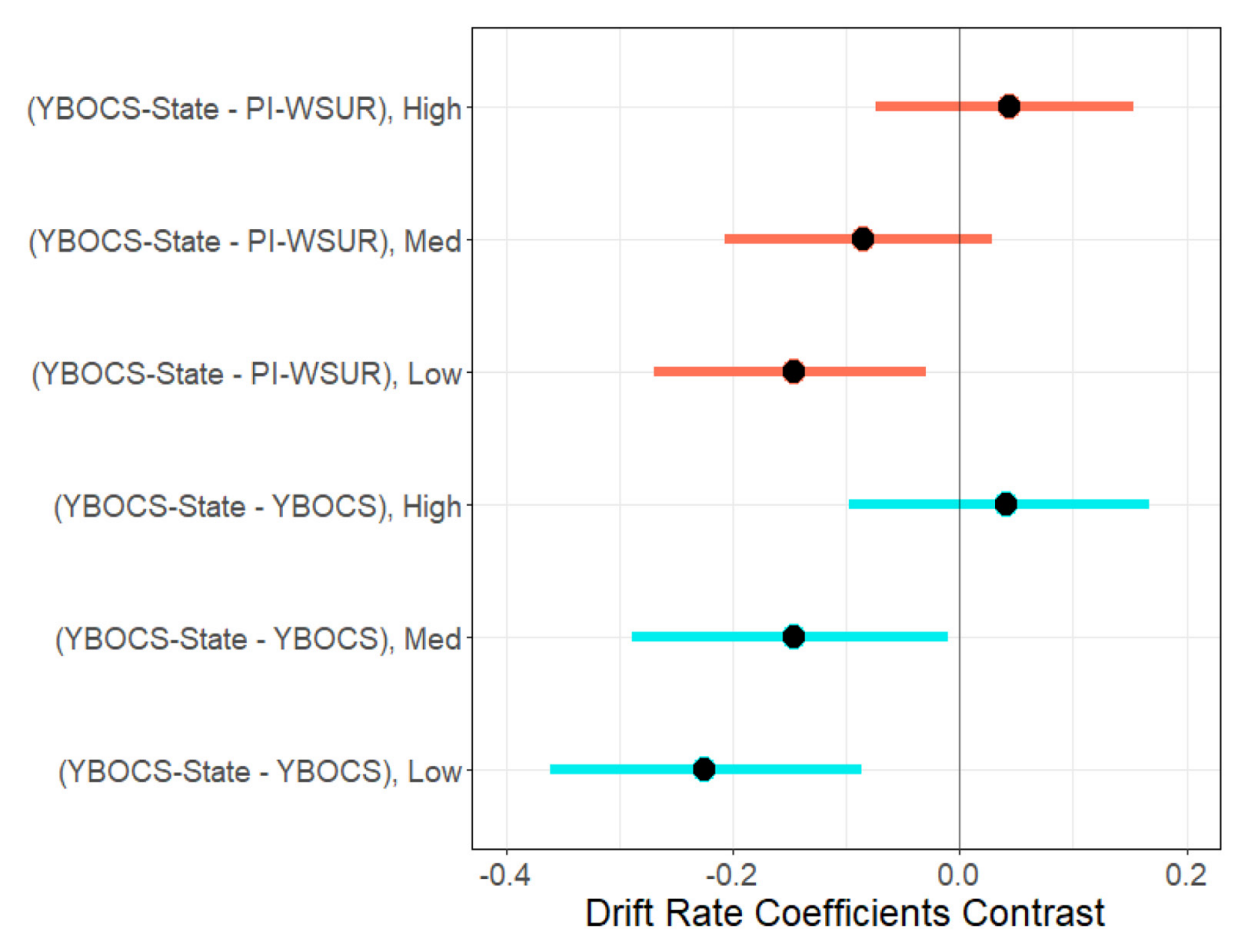
**Contrasts of YBOCS-State versus YBOCS or PI-WSUR Drift Rate Coefficients for Models 3a and 3b (Posterior 95% CI).** “Low” = low uncertainty trials at 45% dot motion coherence; “Med” = medium uncertainty trials at 20% coherence; “High” = high uncertainty trials at 7.5% coherence. Differences (“Contrasts”) between the posterior distribution of the drift rate regression coefficients for YBOCS-State versus PI-WSUR (Model 3a; coral color), and for YBOCS-State versus the standard YBOCS (Model 3b; cyan color) across uncertainty levels. Contrasts were computed by subtracting the corresponding distributions from one another. The median estimate for each contrast’s posterior distribution is represented by a black dot, and the 95% CI is represented by a red line. A contrast is considered significant if the 95% CI does not overlap with 0.

We next tested whether a state-based measure of obsessions and compulsions, the Y-BOCS-SR—State (“YBOCS-State”), better explained these differences than the PI-WSUR or the standard YBOCS. We fit two models to answer this question, one with the PI-WSUR and YBOCS-State as regressors (without the standard YBOCS; Model 3a), and another with the standard YBOCS and the YBOCS-State as regressors (without the PI-WSUR; Model 3b). For Model 3a, YBOCS-State was negatively correlated with drift rate in both Low and Medium uncertainty conditions, and as before the relationships were stronger in easier than in more difficult conditions (Low: β = –0.24 [–0.31, –0.16]; Med: β = –0.11 [–0.19, –0.05]; High: β = 0.02 [–0.05, 0.09]; Difference of Low – Med: β = –0.12 [–0.15, –0.09]; Difference of Med – High: β = –0.14 [–0.16, –0.11]). The PI-WSUR showed a reduced, but still significant, negative effect on drift rate in Low uncertainty trials only (β = –0.09 [ –0.16, –0.01]) and was no longer significant at Medium uncertainty trials. The YBOCS-State effect on drift rate was significantly stronger than that of the PI-WSUR effect at Low uncertainty (Difference of YBOCS-State – YBOCS for Low: β = –0.15 [–0.27, –0.03], Med: β = –0.09 [–0.21, 0.03], High: β = 0.05 [–0.07, 0.16]). For Model 3b, YBOCS-State was negatively correlated with drift rate in both Low and Medium uncertainty conditions (Low: β = –0.25 [–0.34, –0.17]; Med: β = –0.13 [–0.22, –0.05]; High: β = 0.02 [–0.06, 0.10]; Difference of Low – Med: β = –0.12 [–0.15, –0.09]; Difference of Med – High: β = –0.16 [–0.18, –0.13]). Notably, the standard YBOCS showed no relation to drift rate at any uncertainty level. This YBOCS-State effect on drift rate was significantly stronger than that of the standard YBOCS effect at Low and Medium uncertainty (Difference of YBOCS-State – YBOCS for Low: β = –0.23 [–0.37, –0.09], Med: β = –0.15 [–0.29, –0.01], High: β = 0.04 [–0.10, 0.18]). ***Figure 4*** shows the posterior median 95% CI estimates for the effects in Models 3a and 3b. The contrasts at each uncertainty level between the YBOCS-State effects and the YBOCS or the PI-WSUR effects are shown in ***Figure 5***. There were no significant effects of YBOCS, PI-WSUR, or YBOCS-State on decision boundary separation in either model.

We ran an exploratory confirmation of these two models using a larger sample where we included participants regardless of their performance on the dot motion task or the OSPAN task, such that the only exclusion criterion was for the questionnaire catch-items. The rationale here was that, although our performance cut off was standard for OSPAN and nominal for the dot motion task, this may exclude participants with significant symptoms and impairments. This sample had similar demographics (mean age = 38 ± 12, 102 females) as the original sample. Questionnaire total score distributions shifted most for the PI-WSUR (mean = 28, sd = 30, range = 0–140), followed by slighter changes for the YBOCS-State (mean = 5, sd = 6, range = 0–29) and standard YBOCS (mean = 7, sd = 7, range = 0–29). DDM results for both models were similar to our original N = 150 sample in most respects, except that the PI-WSUR effect in Model 3a was now significant at both Low and Medium uncertainty (Low: β = –0.20 [–0.28, –0.11]; Med: β = –0.13 [–0.22, –0.04]; High: β = –0.07 [–0.15, 0.02]) and the difference between YBOCS-State was no longer significantly greater than that of PI-WSUR at any uncertainty level—though it was still significantly greater than the standard YBOCS across all levels. The posterior median 95% CI estimates for the effects with the N = 210 sample can be found in **Supplemental Table S2**.

To test the role of working memory in this context, our fourth model (Model 4) included working memory span scores from the OSPAN task along with all three questionnaires. Drift rate results were largely comparable to Models 3a and 3b for the PI-WSUR, standard YBOCS, and YBOCS-State. YBOCS-State was negatively correlated with drift rate in both Low and Medium uncertainty conditions (Low: β = –0.26 [–0.34, –0.17]; Med: β = –0.14 [–0.22, –0.06]; High: β = 0.02 [–0.06, 0.11]; Difference of Low – Med: β = –0.12 [–0.15, –0.08]; Difference of Med – High: β = –0.17 [–0.19, –0.14]), the PI-WSUR showed a reduced significant negative effect on drift rate in Low uncertainty trials only (β = –0.09 [–0.17, –0.01]) while the standard YBOCS showed no relation to drift rate at any uncertainty level. In this model, the effect of YBOCS-State on drift rate was significantly stronger than the standard YBOCS effect at both Low and Medium uncertainty (Difference of YBOCS-State – YBOCS for Low: β = –0.28 [–0.42, –0.13], Med: β = –0.18 [–0.32, –0.03], High: β = 0.03 [–0.11, 0.18]) and stronger than the PI-WSUR at Low uncertainty (Difference of YBOCS-State – PI-WSUR for Low: β = –0.16 [–0.29, –0.04], Med: β = –0.10 [–0.22, 0.02], High: β = 0.05 [–0.07, 0.17]). Model 4 further revealed that OSPAN score had no significant effect on drift rate nor decision boundary separation at any of the stimulus difficulty levels.

**Figure 6:**
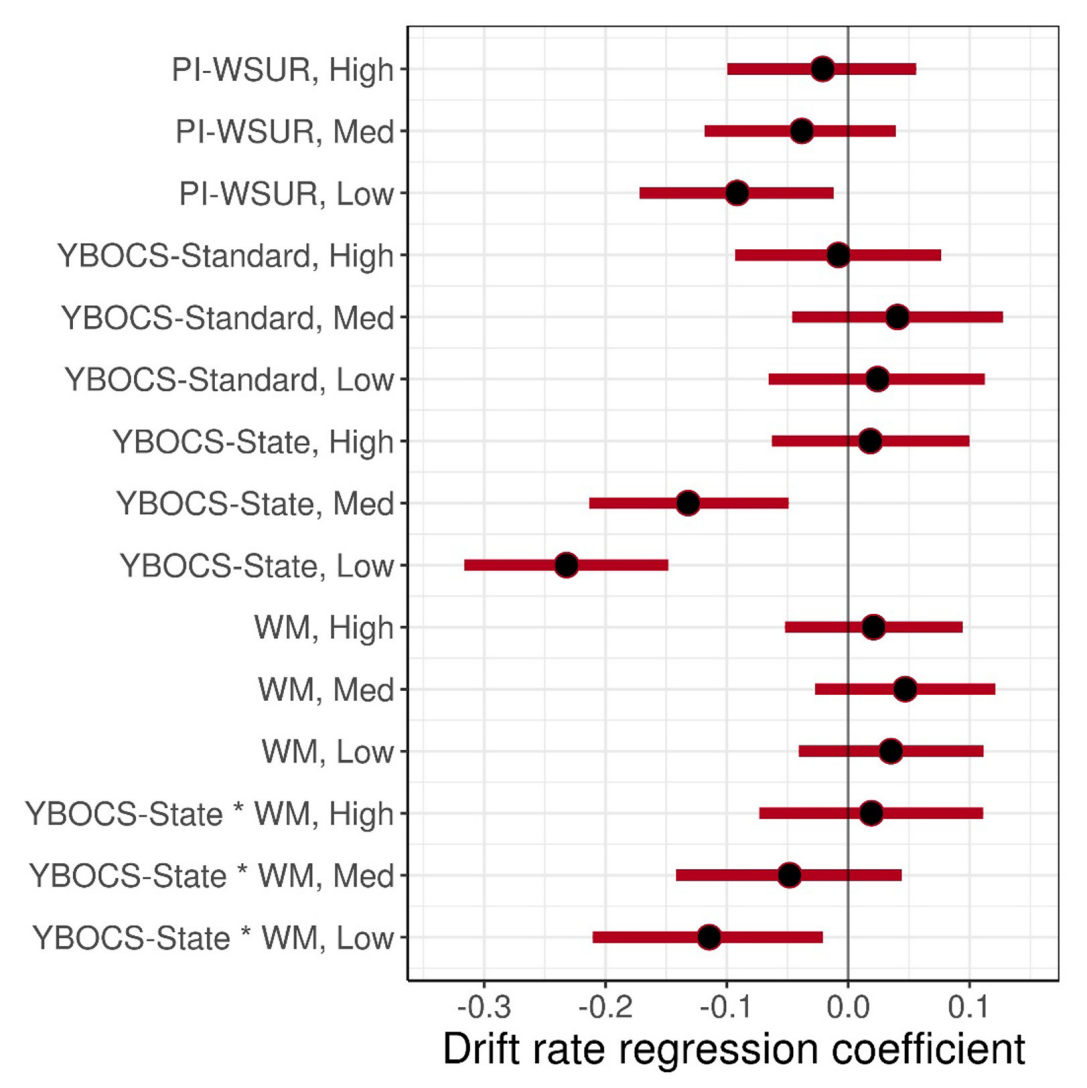
**Posterior Median and 95% CI for Model 5 Regression Coefficients.** Effects of subject-level scores on drift rate. “Low” = low uncertainty trials at 45% dot motion coherence; “Med” = medium uncertainty trials at 20% coherence; “High” = high uncertainty trials at 7.5% coherence. The median estimate for each parameter/regression coefficient’s posterior distribution is represented by a black dot, and the 95% CI is represented by a red line. An effect is considered significant if the 95% CI does not overlap with 0.

To test the hypothesis that a larger working memory span may mitigate the detrimental effect of state-based obsessions/compulsions during the RDMT, our fifth model (Model 5) included the interaction between OSPAN working memory score and YBOCS-State. There was a significant negative interaction effect for Low uncertainty trials, but not Medium or High uncertainty (Low: β = –0.11 [–0.21, –0.02]; Med: β = –0.05 [–0.14, 0.04]; High: β = 0.02 [–0.07, 0.11]; Difference of Low – Med: β = –0.07 [–0.10, –0.03]; Difference of Med – High: β = –0.07 [–0.10, –0.04]), indicating that a larger working memory span actually exacerbated the negative relationship of YBOCS-State score and drift rate, contrary to expectation. Other effects were similar to Model 4. ***Figure 6*** shows the posterior median 95% CI estimates for the effects in this full model.

Given findings in the research literature pertaining to age effects on DDM parameters (Theisen, Lerche, von Krause, & Voss, 2021), we ran an exploratory analysis building off Model 5 to further include the effect of age on both decision threshold and drift rate. Note that while our participants displayed wide variation in age as a whole, we still had relatively few people beyond the cutoff typically found in aging studies (60/65+), and this analysis is truly exploratory. In this model, we also allowed the a priori bias parameter to vary freely. The YBOCS-State effect on drift rate remained significant at both Low and Medium uncertainty, and the PI-WSUR was no longer significant at any uncertainty level. Age was found to have a significant positive effect on drift rate at the Low uncertainty level (Low: β = 0.24 [ 0.17, 0.32]). There was a very slight, but practically negligible, main effect of bias for rightward responses at the subject level (β = –0.02 [–0.05, –0.004]). **Supplemental Table S3** shows posterior median 95% CI estimates for the effects in the exploratory model (“Model 5a”).

As an additional exploratory confirmation of the full model (i.e., Model 5) results, we re-ran the analyses with relaxed exclusion criteria for the OSPAN task. Specifically, we reduced the requirement for accuracy on the processing component (math problems) from 85% to 70% and included participants regardless of their score on the 12-item catch trial. The rationale here, similar to our rationale for our exploratory Model 3a/3b analysis, was that although our initial high cutoff for the math score was standard for this task, it might exclude participants with significant symptoms and impairments. With these changes, the sample size increased to N=177, with similar demographics (mean age = 39 ± 12, 87 females) and OSPAN score distribution (mean = 0.87, range = 0.029–1) as the original sample. Questionnaire total score distributions changed slightly for the PI-WSUR (mean = 24, sd = 24, range = 0–132) and the YBOCS-State (mean = 4, sd = 5, range = 0–25), and remained comparable for the standard YBOCS (mean = 7, sd = 6, range = 0–23). DDM results were similar to our original N = 150 sample in all respects except that: the interaction effect between OSPAN working memory score and YBOCS-State on drift rate was no longer significant at any uncertainty level and the differences between the YBOCS-State and the PI-WSUR effects were likewise no longer significant at any uncertainty level (Difference of YBOCS-State – PI-WSUR for Low: β = –0.04 [–0.16, 0.07], Med: β = –0.03 [–0.14, 0.08], High: β = 0.03 [–0.08, 0.14]). The YBOCS-State effects remained significantly stronger than those of the standard YBOCS at both Low and Medium uncertainty (Difference of YBOCS-State – YBOCS for Low: β = –0.17 [–0.32, –0.03], Med: β = –0.13 [–0.28, –0.006], High: β = –0.02 [–0.16, 0.12]). **Supplemental Table S4** shows posterior median 95% CI estimates for the effects in the exploratory model with N = 177.

## Discussion

In a large general population sample, we examined the relative strength of the relationship between state-level and trait-level OCS and the components of perceptual decision making defined by the drift-diffusion model. Given prior work (Banca et al., 2015; Erhan et al., 2017; Hauser et al., 2017; Marton et al., 2019), we focused specifically on drift rate, a measure of the speed of evidence accumulation over time which reflects the quality or precision of stimulus evidence entering the decision process (Ratcliff & McKoon, 2008). In a simple model including only the PI-WSUR, we found that higher scores on this measure related to slower drift rate on low and medium uncertainty trials. This finding is consistent with a majority of prior studies in both OCD patients and the general population (Banca et al., 2015; Erhan et al., 2017; Hauser et al., 2017; Marton et al., 2019). We then replicated this phenomenon using the Y-BOCS-SR, which is another popular trait measure of OCD symptom severity, thereby demonstrating that this finding was not sensitive to a particular trait-based measure. The enhanced effect of low objective uncertainty contexts aligns with studies showing a similar pattern of impairment for subjective certainty in OCD; for example, Stern and colleagues (2013) found that patients provided greater ratings of subjective uncertainty for low but not higher uncertainty evidence during a probabilistic reasoning task (Stern et al., 2013). Most previous work did not find a relationship between OCD symptoms and decision boundary separation for dot motion decisions (Banca et al., 2015; Erhan et al., 2017; Hauser et al., 2017; Marton et al., 2019), and our results also replicated this pattern.

However, previous work has not tested whether state-based symptom measures better capture perceptual processing differences, leaving it an open question as to whether they represent true underlying long-term deficits. Our analysis revealed that state-based symptoms were substantially negatively related to drift rate, and it further suggested that these effects were at least comparable to, if not larger than, those of trait-based measures. The relationship with state-based symptoms remained after we relaxed our exclusion criteria, which allowed for a broader sample of participants with higher trait severity scores. In this case, although the difference between the Y-BOCS-SR—State and PI-WSUR measures was not significant at any uncertainty level, state effects were still significantly stronger than those measured by the standard Y-BOCS-SR. Perhaps not surprising, we saw a significant correlation between the standard Y-BOCS-SR and Y-BOCS-SR—State. The standard Y-BOCS-SR measures symptoms over the past week and is arguably less of a “trait” measure than the P-WSUR, which asks about general tendencies. However, this does not explain why deficits were significantly more associated with Y-BOCS-SR—State. The less consistent significant differences between the effects of Y-BOCS-SR—State and PI-WSUR suggest that although state effects account for a significant portion of the negative relationship with drift rate, trait effects still carry importance, perhaps especially at lower uncertainty.

A potential concern with this analysis is that although we made minimal changes to the temporal framing of the Y-BOCS-SR to create the state-based measure, its psychometric properties are not known compared to the trait measures, and this may impact the results. Although better understanding the properties of this modified questionnaire is an important topic for future work, it should be noted that we found that it *better* captured the relationship with drift rate, not worse, speaking against reliability concerns. Indeed, there is possibly a greater concern regarding the trait-based measures. Although both measures are well-established and the same or similar measures were used in previous studies of perceptual deficits related to OCD symptoms, asking individuals to comment on past behavior can be unreliable and can potentially result in trait effects being misestimated. It would be of interest to repeat this work while using journaling or more modern smartphone-based momentary assessment techniques that measure symptoms in the wild repeatedly over long time scales.

As previously noted, trait-based effects on evidence accumulation have been replicated in both patient and non-patient samples (Banca et al., 2015; Erhan et al., 2017; Hauser et al., 2017; Marton et al., 2019), suggesting that this phenomenon is not sensitive to formal diagnostic classification. Even so, one limitation worth acknowledging is that our findings of significant state-based effects are in a non-clinical general population sample with relatively lower symptom scores than would be expected in OCD patients. Although we suspect the effect of state-based symptoms might be even greater in a clinical OCD sample, this remains a question for future work. Another limitation is the lack of specificity of our state results. State-based OC symptoms are also likely related to other factors that could drive deficits, such as general nervousness and unease. It would be fruitful to further test the specificity of these results against other state measures such as the STAI-S (Spielberger, Gorsuch, Lushene, Vagg, & Jacobs, 1983).

An obvious question is how one might interpret the results obtained here from a more mechanistic perspective of the disorder. We attempt to avoid drawing strong conclusions from correlational data, but we do offer some speculation that can be tested in future work. A particularly parsimonious possibility is that state-based symptoms drive drift rate deficits by tying up attentional resources with processing the obsessions and mental compulsions which define them. Their impact then fluctuates over time as they wax and wane. Clinical models have also suggested that attentional bias towards threat, as well as maladaptive attentive-regulatory strategies towards negative or unwanted thoughts, may play a causal role in generating symptoms (Muller & Roberts, 2005; Adrian Wells, 1997; Adrian Wells, 2009; Adrian Wells & Matthews, 1994). These biases and modes of processing themselves may tie up attentional resources in addition to obsessions and compulsions. Of course, the general hypothesis of off-tasking processing resulting in secondary performance deficits has previously been articulated by others, and we are not the first to suggest it (Abramovitch et al., 2012; Moritz et al., 2012). Testing it in the current context in a causal fashion will require the creation of sophisticated new experimental paradigms that can dynamically manipulate attention to symptoms during perceptual decision making. Although such a feat will by no means be trivial, it should be noted that existing work has already demonstrated how one can study some of the separate pieces of the larger puzzle. First, the relationship between attention and drift rate during perceptual decision making has been demonstrated in two ways (Tavares, Perona, & Rangel, 2017): by passively measuring within-trial attentional priority using eye-tracking, and by differentially manipulating exogeneous attention to different options under consideration by assigning each a different minimum fixation time. Second, other work has also shown how symptoms could be provoked in the lab using idiosyncratic threat stimuli (Nakao, Okada, & Kanba, 2014). A future hybrid task could track and manipulate the amount of attention devoted to threat stimuli versus perceptual evidence in a concurrent task. Of note, evidence accumulation improvements in OCD patients have already been demonstrated using incentive manipulation for speed over accuracy ^13^. This shows that impairments can indeed be rescued through behavioral intervention, although changes in the incentive structure could drive improvement through a number of different mechanisms, attention being only one of them.

We began to ask a similar question here as it relates to working memory and did not find evidence that a larger working memory capacity could buttress state-based effects, speaking perhaps against an attention-based explanation. However, we note that while attention and working memory are related, they are not the same. With regard to working memory, our results indicated the opposite—that a larger capacity may further exacerbate state-based effects on evidence accumulation. One possible interpretation could be that individuals with high working memory capacity actually have more baseline resources to put towards the monitoring of—and thus ultimate exacerbation of—said symptoms (Cohen & Calamari, 2004; Adrian Wells, 2009; Adrian Wells & Matthews, 1994). To index working memory we used the OSPAN, which focuses on verbal memory and is largely unrelated to perceptual performance. This choice was purposeful and has both advantages and an important disadvantage. With regard to the advantages, first, it protects against a potential confound resulting from shared variance in performance between a perceptual working memory task and the dot motion task that is unrelated to memory ability. Second, most obsessions and many compulsions are verbal in nature (American Psychiatric Association, 2013; Collaton & Purdon, 2015), and differences in verbal working memory may play a role for this reason. On the other hand, differences in visual working memory also likely play an important role, as the impairments are perceptual in nature. It would thus be of interest to repeat asking about the role of working memory using a perceptual working memory measure.

In summary, we found that state-based symptoms explained substantial variance in the overall relationship between obsessive-compulsive symptoms and drift rate impairments in perceptual decision making. These effects were not consistently moderated by differences in working memory capacity. This study illustrates the potential importance of asking about state effects in other work which focuses on relationships between psychopathology and parameters of computational models.

## Data Accessibility Statement

Our raw data (dot motion task, OSPAN task, questionnaires, age/gender demographic data) and R code for running the main drift-diffusion models (Models 1, 2, 3a, 3b, 4, 5) for the N = 150 and N = 177 samples have been made publicly available at: *https://osf.io/vha5r/?view_only=8d417853f79f4f01818161edf6510c0b*.

## Additional files

The additional files for this article can be found as follows:

10.5334/cpsy.87.s1 Supplement. Supplementary Methods and Results.

10.5334/cpsy.87.s2 Supplemental Table S1. Posterior Median and 95% CI for Regression Coefficients in Each Model.

10.5334/cpsy.87.s3 Supplemental Table S2. Posterior Median and 95% CI for Regression Coefficients in Models 3a/3b for the N=210 Relaxed RDM and OSPAN Performance Sample.

10.5334/cpsy.87.s4 Supplemental Table S3. Posterior Median and 95% CI for Regression Coefficients in Model 5a for the N=150 Sample.

10.5334/cpsy.87.s5 Supplemental Table S4. Posterior Median and 95% CI for Regression Coefficients in Model 5 for the N=177 Relaxed OSPAN-Criteria Sample.

## References

[1] Abramovitch, A., Dar, R., Hermesh, H., & Schweiger, A. (2012). Comparative neuropsychology of adult obsessive-compulsive disorder and attention deficit/hyperactivity disorder: implications for a novel executive overload model of OCD. J Neuropsychol, 6(2), 161–191. DOI: 10.1111/j.1748-6653.2011.02021.x 22257360

[2] Abramowitz, J. S., Taylor, S., & McKay, D. (2009). Obsessive-compulsive disorder. The Lancet, 374(9688), 491–499. DOI: 10.1016/S0140-6736(09)60240-3 19665647

[3] American Psychiatric Association. (2013). Diagnostic and Statistical Manual of Mental Disorders. American Psychiatric Association. DOI: 10.1176/appi.books.9780890425596

[4] Baer, L., Brown-Beasley, M. W., Sorce, J., & Henriques, A. I. (1993). Computer-assisted telephone administration of a structured interview for obsessive-compulsive disorder. American Journal of Psychiatry, 150(11), 1737–1738. DOI: 10.1176/ajp.150.11.1737 8214187

[5] Banca, P., Vestergaard, M. D., Rankov, V., Baek, K., Mitchell, S., Lapa, T., … Voon, V. (2015). Evidence accumulation in obsessive-compulsive disorder: the role of uncertainty and monetary reward on perceptual decision-making thresholds. Neuropsychopharmacology, 40(5), 1192–1202. DOI: 10.1038/npp.2014.303 25425323 PMC4349497

[6] Bogacz, R., Brown, E., Moehlis, J., Holmes, P., & Cohen, J. D. (2006). The physics of optimal decision making: A formal analysis of models of performance in two-alternative forced-choice tasks. Psychological Review, 113(4), 700–765. DOI: 10.1037/0033-295X.113.4.700 17014301

[7] Buhrmester, M., Kwang, T., & Gosling, S. D. (2016). Amazon’s Mechanical Turk: A new source of inexpensive, yet high-quality data? In Methodological issues and strategies in clinical research (4th ed.) (pp. 133–139). American Psychological Association. DOI: 10.1037/14805-009 26162106

[8] Burns, G. L., Keortge, S. G., Formea, G. M., & Sternberger, L. G. (1996). Revision of the Padua Inventory of obsessive compulsive disorder symptoms: Distinctions between worry, obsessions, and compulsions. Behaviour Research and Therapy, 34(2), 163–173. DOI: 10.1016/0005-7967(95)00035-6 8741724

[9] Cohen, R. J., & Calamari, J. E. (2004). Thought-Focused Attention and Obsessive–Compulsive Symptoms: An Evaluation of Cognitive Self-Consciousness in a Nonclinical Sample. Cognitive Therapy and Research, 28(4), 457–471. DOI: 10.1023/B:COTR.0000045558.75538.ff

[10] Collaton, J., & Purdon, C. (2015). Verbalizations during compulsions in obsessive-compulsive disorder. Journal of Obsessive-Compulsive and Related Disorders, 7, 49–53. DOI: 10.1016/j.jocrd.2015.10.004

[11] Conway, A. R., Kane, M. J., Bunting, M. F., Hambrick, D. Z., Wilhelm, O., & Engle, R. W. (2005). Working memory span tasks: A methodological review and user’s guide. Psychonomic Bulletin & Review, 12, 769–786. DOI: 10.3758/BF03196772 16523997

[12] Crump, M. J. C., McDonnell, J. V., & Gureckis, T. M. (2013). Evaluating Amazon’s Mechanical Turk as a Tool for Experimental Behavioral Research. PLoS ONE, 8(3), e57410. DOI: 10.1371/journal.pone.0057410 23516406 PMC3596391

[13] Erhan, C., & Balcı, F. (2017). Obsessive compulsive features predict cautious decision strategies. Quarterly Journal of Experimental Psychology, 70(1), 179–190. DOI: 10.1080/17470218.2015.1130070 26652727

[14] Erhan, C., Bulut, G. Ç., Gökçe, S., Ozbas, D., Turkakin, E., Dursun, O. B., … Balcı, F. (2017). Disrupted latent decision processes in medication-free pediatric OCD patients. Journal of Affective Disorders, 207, 32–37. DOI: 10.1016/j.jad.2016.09.011 27690351

[15] Federici, A., Summerfeldt, L. J., Harrington, J. L., McCabe, R. E., Purdon, C. L., Rowa, K., & Antony, M. M. (2010). Consistency between self-report and clinician-administered versions of the Yale-Brown Obsessive–Compulsive Scale. Journal of Anxiety Disorders, 24(7), 729–733. DOI: 10.1016/j.janxdis.2010.05.005 20561767

[16] Foa, E. B., Mathews, A., Abramowitz, J. S., Amir, N., Przeworski, A., Riggs, D. S., … Alley, A. (2003). Do Patients with Obsessive–Compulsive Disorder Have Deficits in Decision-Making? Cognitive Therapy and Research, 27(4), 431–445. DOI: 10.1023/A:1025424530644

[17] Gillan, C. M., Kosinski, M., Whelan, R., Phelps, E. A., & Daw, N. D. (2016). Characterizing a psychiatric symptom dimension related to deficits in goal-directed control. eLife, 5, e11305. DOI: 10.7554/eLife.11305 26928075 PMC4786435

[18] Gold, J. I., & Shadlen, M. N. (2007). The Neural Basis of Decision Making. Annual Review of Neuroscience, 30(1), 535–574. DOI: 10.1146/annurev.neuro.29.051605.113038 17600525

[19] Goodman, W. K. (1989). The Yale-Brown Obsessive Compulsive Scale. Archives of General Psychiatry, 46(11), 1006–1011. DOI: 10.1001/archpsyc.1989.01810110048007 2684084

[20] Hauser, T. U., Allen, M., Rees, G., & Dolan, R. J. (2017). Metacognitive impairments extend perceptual decision making weaknesses in compulsivity. Scientific Reports, 7(1), 6614. DOI: 10.1038/s41598-017-06116-z 28747627 PMC5529539

[21] Heekeren, H. R., Marrett, S., & Ungerleider, L. G. (2008). The neural systems that mediate human perceptual decision making. Nature Reviews Neuroscience, 9(6), 467–479. DOI: 10.1038/nrn2374 18464792

[22] Lerche, V., & Voss, A. (2016). Model Complexity in Diffusion Modeling: Benefits of Making the Model More Parsimonious. Frontiers in Psychology, 7, 1324–1324. DOI: 10.3389/fpsyg.2016.01324 27679585 PMC5020081

[23] Marton, T., Samuels, J., Nestadt, P., Krasnow, J., Wang, Y., Shuler, M., … Nestadt, G. (2019). Validating a dimension of doubt in decision-making: A proposed endophenotype for obsessive-compulsive disorder. PLoS ONE, 14(6), e0218182–e0218182. DOI: 10.1371/journal.pone.0218182 31194808 PMC6564001

[24] Moritz, S., Hottenrott, B., Jelinek, L., Brooks, A. M., & Scheurich, A. (2012). Effects of Obsessive-Compulsive Symptoms on Neuropsychological Test Performance: Complicating an Already Complicated Story. The Clinical Neuropsychologist, 26(1), 31–44. DOI: 10.1080/13854046.2011.639311 22166079

[25] Muller, J., & Roberts, J. (2005). Memory and attention in Obsessive-Compulsive Disorder: A review. Journal of Anxiety Disorders, 19, 1–28. DOI: 10.1016/j.janxdis.2003.12.001 15488365

[26] Nakao, T., Okada, K., & Kanba, S. (2014). Neurobiological model of obsessive-compulsive disorder: Evidence from recent neuropsychological and neuroimaging findings. Psychiatry and Clinical Neurosciences, 68(8), 587–605. DOI: 10.1111/pcn.12195 24762196

[27] Newsome, W. T., Britten, K. H., & Movshon, J. A. (1989). Neuronal correlates of a perceptual decision. Nature, 341(6237), 52–54. DOI: 10.1038/341052a0 2770878

[28] Pushkarskaya, H., Tolin, D., Ruderman, L., Kirshenbaum, A., Kelly, J. M., Pittenger, C., & Levy, I. (2015). Decision-making under uncertainty in obsessive–compulsive disorder. Journal of Psychiatric Research, 69, 166–173. DOI: 10.1016/j.jpsychires.2015.08.011 26343609 PMC4562025

[29] R Core Team. (2019). R: A language and environment for statistical computing. In: Vienna, Austria: R Foundation for Statistical Computing. https://www.R-project.org/

[30] Ratcliff, R. (1978). A theory of memory retrieval. Psychological Review, 85(2), 59–108. DOI: 10.1037/0033-295X.85.2.59

[31] Ratcliff, R., & McKoon, G. (2008). The Diffusion Decision Model: Theory and Data for Two-Choice Decision Tasks. Neural Computation, 20(4), 873–922. DOI: 10.1162/neco.2008.12-06-420 18085991 PMC2474742

[32] Ratcliff, R., & Rouder, J. N. (1998). Modeling Response Times for Two-Choice Decisions. Psychological Science, 9(5), 347–356. DOI: 10.1111/1467-9280.00067

[33] Reed, G. F. (1976). Indecisiveness in Obsessional-Compulsive Disorder. British Journal of Social and Clinical Psychology, 15(4), 443–445. DOI: 10.1111/j.2044-8260.1976.tb00059.x 1000152

[34] Rubio-Aparicio, M., Núñez-Núñez, R. M., Sánchez-Meca, J., López-Pina, J., Marín-Martínez, F., & López-López, J. A. (2017). A Reliability Generalization Meta-Analysis of the Padua Inventory of Obsessions and Compulsions. The Spanish Journal of Psychology, 20, e70. DOI: 10.1017/sjp.2017.65 29198230

[35] Sachdev, P. S., & Malhi, G. S. (2005). Obsessive–Compulsive Behaviour: A Disorder of Decision-Making. Australian & New Zealand Journal of Psychiatry, 39(9), 757–763. DOI: 10.1080/j.1440-1614.2005.01680.x 16168033

[36] Shadlen, M. N., & Newsome, W. T. (1996). Motion perception: seeing and deciding. Proceedings of the National Academy of Sciences, 93(2), 628–633. DOI: 10.1073/pnas.93.2.628 PMC40102 8570606

[37] Smith, P. L., & Ratcliff, R. (2004). Psychology and neurobiology of simple decisions. Trends in Neurosciences, 27(3), 161–168. DOI: 10.1016/j.tins.2004.01.006 15036882

[38] Solway, A., Lin, Z., & Vinaik, E. (2021). Transfer of information across repeated decisions in general and in obsessive–compulsive disorder. Proceedings of the National Academy of Sciences, 118(1), e2014271118. DOI: 10.1073/pnas.2014271117 PMC7817149 33443150

[39] Spielberger, C., Gorsuch, R., Lushene, R., Vagg, P. R., & Jacobs, G. (1983). Manual for the State-Trait Anxiety Inventory (Form Y1 – Y2) (Vol. IV). DOI: 10.1037/t06496-000

[40] Stan Development Team. (2018). RStan: the R interface to Stan. R package version 2.18.2. http://mc-stan.org. In.

[41] Steketee, G., Frost, R., & Bogart, K. (1996). The Yale-Brown Obsessive Compulsive Scale: Interview versus self-report. Behaviour Research and Therapy, 34(8), 675–684. DOI: 10.1016/0005-7967(96)00036-8 8870295

[42] Stern, E. R., Welsh, R. C., Gonzalez, R., Fitzgerald, K. D., Abelson, J. L., & Taylor, S. F. (2013). Subjective uncertainty and limbic hyperactivation in obsessive-compulsive disorder. Human brain mapping, 34(8), 1956–1970. DOI: 10.1002/hbm.22038 22461182 PMC5289818

[43] Tavares, G., Perona, P., & Rangel, A. (2017). The Attentional Drift Diffusion Model of Simple Perceptual Decision-Making. Frontiers in Neuroscience, 11. DOI: 10.3389/fnins.2017.00468 PMC5573732 28894413

[44] Theisen, M., Lerche, V., von Krause, M., & Voss, A. (2021). Age differences in diffusion model parameters: a meta-analysis. Psychological Research, 85(5), 2012–2021. DOI: 10.1007/s00426-020-01371-8 32535699 PMC8289776

[45] Torres, A. R., Prince, M. J., Bebbington, P. E., Bhugra, D., Brugha, T. S., Farrell, M., … Singleton, N. (2006). Obsessive-Compulsive Disorder: Prevalence, Comorbidity, Impact, and Help-Seeking in the British National Psychiatric Morbidity Survey of 2000. American Journal of Psychiatry, 163(11), 1978–1985. DOI: 10.1176/ajp.2006.163.11.1978 17074950

[46] Townsend, J. T., & Ashby, F. G. (1983). The Stochastic Modeling of Elementary Psychological Processes. New York: Cambridge University Press.

[47] Turner, M. L., & Engle, R. W. (1989). Is working memory capacity task dependent? Journal of Memory and Language, 28(2), 127–154. DOI: 10.1016/0749-596X(89)90040-5

[48] Veale, D., & Roberts, A. (2014). Obsessive-compulsive disorder. BMJ, 348(apr07 6), g2183–g2183. DOI: 10.1136/bmj.g2183 24709802

[49] Wells, A. (1997). Cognitive Therapy of Anxiety Disorders: A Practice Manual and Conceptual Guide. Chichester: Wiley.

[50] Wells, A. (2009). Metacognitive therapy for anxiety and depression. New York, NY: The Guildford Press.

[51] Wells, A., & Matthews, G. (1994). Attention and emotion: A clinical perspective. Hillsdale, NJ, US: Lawrence Erlbaum Associates, Inc.

[52] White, C. N., Ratcliff, R., Vasey, M. W., & McKoon, G. (2010). Using diffusion models to understand clinical disorders. Journal of mathematical psychology, 54(1), 39–52. DOI: 10.1016/j.jmp.2010.01.004 20431690 PMC2859713

