# Supplementary material for: Perceptual Decision Impairments Linked to Obsessive-Compulsive Symptoms are Substantially Driven by State-Based Effects
Source: Comput Psychiatr. 2022 May 12;6(1):79–95. doi: 10.5334/cpsy.87 (PMC11104319; doi:10.5334/cpsy.87)
Supplement: Supplement. — Supplementary Methods and Results. [file cpsy-6-1-87-s1.pdf]

Supplementary Methods and Results to accompany—

Perceptual decision impairments linked to obsessive-compulsive symptoms are substantially driven by state-based effects

Claire M. Kaplan[1]
Alec Solway [1,2]

[1] Department of Psychology and [2] Program in Neuroscience and Cognitive Science, University of Maryland-College Park, MD 20742 USA.

Supplemental Table S1. ***Posterior Median and 95% CI for Regression Coefficients in Each Model.***

The central 95% credible interval (CI) is represented as the range between the numbers in the "Lower" and "Upper" columns. Effects are considered significant when the 95% CI does not include 0.
(see attached excel file for Supplemental Table S1)

Supplemental Table S2. ***Posterior Median and 95% CI for Regression Coefficients in Models 3a/3b for the N=210 Relaxed RDM and OSPAN Performance Sample.***

The central 95% credible interval (CI) is represented as the range between the numbers in the "Lower" and "Upper" columns. Effects are considered significant when the 95% CI does not include 0.
(see attached excel file for Supplemental Table S2)

Supplemental Table S3. ***Posterior Median and 95% CI for Regression Coefficients in Model 5a for the N=150 Sample.***

The central 95% credible interval (CI) is represented as the range between the numbers in the "Lower" and "Upper" columns. Effects are considered significant when the 95% CI does not include 0.
(see attached excel file for Supplemental Table S3)

Supplemental Table S4. ***Posterior Median and 95% CI for Regression Coefficients in Model 5 for the N=177 Relaxed OSPAN-Criteria Sample.***

The central 95% credible interval (CI) is represented as the range between the numbers in the "Lower" and "Upper" columns. Effects are considered significant when the 95% CI does not include 0.
(see attached excel file for Supplemental Table S4)

SUPPLEMENTAL METHODS – Additional Details

Participants and Tasks

Potential participants first completed a training phase that consisted of written instructions followed by a brief practice round for each task. They were then required to correctly answer three comprehension questions pertaining to basic task rules in order to participate in the main experiment.

The training phase and associated comprehension questions were always administered first, followed by the two tasks—a perceptual decision-making task (random-dot motion task; RDMT) and a working memory task (operation span; OSPAN)—presented in randomized order, and finally several self-report questionnaires. Of those questionnaires, the state measure of obsessive-compulsive symptoms (Y-BOCS-SR—State; see *Y-BOCS-SR Questionnaires* below) was always presented first, right after both tasks were completed. The order of the remaining questionnaires was randomized.

Participants were sequentially excluded from analyses in the following order: 1) if their total accuracy on the dot motion task across all conditions was below 55% (n=69), 2) if their accuracy on the processing component of the OSPAN (mathematics problems) was below 85% (Conway et al., 2005) (n=16), or 3) if they answered either of two questionnaire catch-items incorrectly (n=25). Catch questions appeared in the Y-BOCS-SR—State and the Padua Inventory and said e.g., "Upon seeing this question, please select 'Very much' for your answer." Further, to reduce instances of cheating on the OSPAN (e.g., writing down target items to assist later recall), a 12-item catch trial was presented at the end of the task; participants who scored a 12 out of 12 were excluded from analyses

(n=16). In total, 126 out of 276 (45%) subjects who submitted data were excluded from the study.

Random Dot Motion Task

In each trial of the random-dot motion task (RDMT), participants were shown a dynamic kinematogram of 30 small white dots on a black background that repositioned to a new location on each frame. The kinematogram was presented in a circular aperture (diameter of 350 pixels) centered in the middle of the screen, and coherent dots (randomly chosen on each frame) moved at a speed of 10 pixels per frame. The remaining dots appeared at random. Trials consisted of varying levels of motion coherence either leftward or rightward – the larger the level of motion coherence, the greater the proportion of dots moving unambiguously in one direction. Participants were asked to report the primary direction of motion by pressing a key on the keyboard ("Q" for left; "P" for right). The stimulus was displayed until a keyboard response was given, with no time limit for responses (ITI = 500ms). Coherence levels were set to 7.5% ("High uncertainty"), 20% ("Medium uncertainty"), and 45% ("Low uncertainty"), and presented in random order across trials. Participants completed three blocks of 120 trials each.

Operation-SPAN Working Memory Task

Participants completed a computerized version of the OSPAN task (Turner & Engle, 1989). Within each trial, participants were shown a series of alternating simple math problems and single letters, wherein a single math problem followed by a single letter is referred to as an equation-letter pair. Answers were supplied for the math problems (e.g., 2x9 – 9 = 9) and participants were asked to verify whether the equation was correct by pressing "Q" for

false or “P” for true, within a time limit (20 secs). Equations were compiled randomly according to the standard OSPAN format, with equal correct and incorrect answers. Each letter was presented for 800ms in the center of the screen, and was randomly drawn without replacement from a pool of 12 non-vowel letters for each trial. Trials ranged in length from 3 to 7 successively presented equation-letter pairs. At the end of each trial, participants were prompted to recall the letters in correct order by typing each letter into a blank row from top to bottom. Three trials of each length (3-7) were presented, for a total of 15 trials. A catch trial consisting of 12 equation-letter pairs was always presented last, for exclusion purposes only. Individual working memory span scores were calculated using the partial-credit unit scoring method (for description and reasoning, see Conway et al., 2005).

Y-BOCS-SR Questionnaires

The Y-BOCS-SR was modified to ask specifically about the obsessions and compulsions experienced during task performance in the experiment (Y-BOCS-SR—State). Changes included the following. First, we added a time specifier to precede each question in both versions of the YBOCS-SR to remind participants that we were asking about symptoms experienced “during the dots and math/letters tasks” only or “over the past week”. At this point, participants were already familiarized to refer to the RDMT as the “Dots Task” and to the OSPAN as the “Math/Letters Task.” We also changed the answer options for both versions for items 1 and 6 of this scale to represent time in terms of a percentage of the total probed duration (the original version presents options in terms of number of hours in an 8 hour day span). Finally, we added the specifier of “intrusions” as an interchangeable

term for obsessions, and the specifier of "rituals" as an interchangeable term for compulsions; given that our participants completed the experiment online, remotely, and had diverse backgrounds, this was done to help them more easily conceptualize symptoms clinically known as "obsessions" and "compulsions"

Stimuli and Apparatus

Task stimuli were generated using plugins from jsPsych (https://www.jspsych.org), which is a JavaScript library for designing and running behavioral experiments in web browsers online. The random-dot motion task used in the current study was modified from publicly available code online for the jsPsych-RDK plugin (https://github.com/vrsivananda/RDK).

To interact with the MTurk service (e.g., post our study, recruit and pay participants), we utilized the psiTurk toolbox (https://psiturk.org), which is an open source resource for interfacing with MTurk in the context of behavioral data collection.

Drift-Diffusion Models

Bayesian inference was performed for each model using Stan (Stan Development Team). For all models, we set the upper boundary to be the correct response and the lower boundary to be the incorrect response. Drift rate towards the correct boundary is represented by a positive value, while drift rate towards the incorrect decision is represented by a negative value.

In specifying each model, the boundary separation (alpha) and drift rate (delta) parameters included regression formulas comprising: a subject-level intercept, a mean group condition-level intercept, and a group condition-level regression coefficients for each

of the respective questionnaire scores and interactions of interest. For example, the regression equations for alpha and delta in Model 1 were as follows:

alpha = subject_alpha[s] + condition_alpha[c] + alpha_coef[c]*score[s]

delta = subject_delta[s] + condition_delta[c] + delta_coef[c]*score[s]

where [s] is the subject, [c] is the stimulus coherence condition level (low, medium, or high uncertainty), and score is the z-scored total on the PI-WSUR questionnaire.

Model 2 followed the same specifications as above, except score was the z-scored total on the standard Y-BOCS-SR questionnaire. Models 3a and 3b specified two condition-level regression coefficients each (3a comprising the PI-WSUR score and the Y-BOCS-SR—State score; 3b comprising the standard Y-BOCS-SR score and the Y-BOCS-SR—State score). Model 4 included a fourth condition-level regression coefficient for the OSPAN working memory score. Model 5 included a final condition-level regression coefficient for the interaction of Y-BOCS-SR—State score * OSPAN working memory score.

For the subject-level parameters for boundary separation (subject_alpha) and drift rate (subject_delta), we set hierarchical prior distributions of ~normal(0, σ), where σ in turn had a prior of ~normal(0, 20). On the condition-level parameter for boundary separation (condition_alpha), we set a prior of ~normal(1, 20) (the boundary separation is biased positive, but the variance is high and the prior is only weakly informative). This parameter, as well as the σ's, were set to have a lower bound of 0. On the condition-level parameter for drift rate (condition_delta), we set an unbiased prior of ~normal(0, 20). All condition-level regression coefficients for boundary separation and drift rate had unbiased priors of ~normal(0, 20).

Non-decision time (tau) was allowed to vary by subject only. We set a hierarchical prior of ~normal(μ, σ) on tau , in which μ had a prior of ~normal(0.5, 5) and σ had a prior of ~normal(0, 5).

Finally, given that left/right responses in the RDMT were balanced, the starting point bias was fixed to 0.5 in all models, representing no starting bias in either direction.

For all models, four independent Markov chains were run for 8,000 iterations, with the first 1,000 samples discarded as burn-in. Convergence was assessed with visual inspection of the Markov chains and by computing the R-hat Gelman-Rubin statistic where successful convergence is indicated by values <1.1.
